# A rabies-related lyssavirus from a *Nycticeinops schlieffeni* bat with neurological signs, South Africa

**DOI:** 10.1128/MRA.00621-23

**Published:** 2023-10-06

**Authors:** Natalie Viljoen, Arshad Ismail, Jacqueline Weyer, Wanda Markotter

**Affiliations:** 1 Centre for Viral Zoonoses, Department of Medical Virology, University of Pretoria, Pretoria, South Africa; 2 Centre for Emerging Zoonotic and Parasitic Diseases, National Institute for Communicable Disease of the National Health Laboratory Service, Sandringham, South Africa; 3 Sequencing Core Facility, National Institute for Communicable Diseases of the National Health Laboratory Service, Sandringham, South Africa; 4 Department of Biochemistry and Microbiology, Faculty of Science, Engineering and Agriculture, University of Venda, Thohoyandou, South Africa; 5 Institute for Water and Wastewater Technology, Durban University of Technology, Durban, South Africa; 6 Department of Microbiology and Infectious Diseases, Faculty of Health Sciences, University of Witwatersrand, Johannesburg, South Africa; Katholieke Universiteit Leuven, Leuven, Belgium

**Keywords:** *Lyssavirus*, rabies, South Africa, bat, surveillance, neurological

## Abstract

We report the coding-complete sequence of a lyssavirus, provisionally designated Phala bat lyssavirus (PBLV), characterized using a metagenomics approach. PBLV was identified in a *Nycticeinops schlieffeni* bat that exhibited neurological signs and died within 24 hours of admission to a wildlife rehabilitation center in Phalaborwa, South Africa.

## ANNOUNCEMENT

Bats are considered to be important hosts for viruses that belong to the genus *Lyssavirus*, subfamily *Alpharhabdovirinae*, family *Rhaboviridae* (1, 2). As part of a disease ecology of zoonotic pathogens in bats study, a bat collected in Phalaborwa, South Africa (coordinates: −23.943190 and 31.128990; laboratory number: UP14561) that displayed neurological signs and died on 7 September 2021 within 24 hours of admission to a wildlife rehabilitation center was submitted for investigation. Brain material from a *Nycticeinops schlieffeni* bat, confirmed by DNA barcoding (*CytB*, *COI*, and *12S rRNA* genes) (3 – 7), was homogenized, and nucleic acids were extracted using the NucleoMag VET RNA/DNA kit (Macherey-Nagel). A lyssavirus quantitative reverse-transcriptase PCR (8) with modifications to the probe (Table 1) was positive and was confirmed by partial nucleoprotein gene amplification (9). Double-stranded complementary DNA was prepared from total RNA using Superscript IV (Thermo Fisher Scientific) and random hexamer primers (Integrated DNA Technologies) followed by degradation of the RNA strand using 10U RNase H (Ambion) and second-strand synthesis using 5U Klenow 3′−5′ Exo-minus DNA polymerase (Thermo Fisher Scientific) (10) in a single step. DNA was purified using the MinElute PCR purification kit (Qiagen) and quantified using a Qubit fluorometer (Thermo Fisher Scientific). Paired-end libraries (2 × 150 bp) were prepared using the Nextera DNA flex preparation kit (Illumina) according to the manufacturer’s instructions, and sequencing was performed on 30-ng cDNA on a NextSeq 2000 instrument (Illumina).

**TABLE 1:** Primers and probes used for amplification and sequencing

Application	Primer/probe name	Primer/probe sequence (5′−3′)^*a*^:	Position on reference	Reference
Cytochrome B barcoding PCR	LGL 765	GAAAAACCAYCGTTGTWATTCAACT	14,710–14,734^*b*^	(3)
	LGL 766	GTTTAATTAGAATYTYAGCTTTGGG	15,989–16,014^*b*^	(4)
12S rRNA barcoding PCR	12SU1230M2-CH	GCACTGAAAATGCYTAGATG	607–625^*b*^	(5)
	12SL2226M1	CAGTAYGCTTACCTTGTTACGAC	1,559–1,581^*b*^	(6)
Cytochrome C oxidase subunit I gene barcoding PCR	LCO1490	GGTCAACAAATCATAAAGATATTGG	5,931–5,950^*b*^	(7)
	HCO2198	TAAACTTCAGGGTGACCAAAAAATCA	6,609–6,634^*b*^	
*Lyssavirus* partial NP RT-PCR^*d*^	lys001	ACGCTTAACGAMAAA	1–15^*c*^	(9)
	550B	GTRCTCCARTTAGCRCACAT	647–666^*c*^	
*Lyssavirus* screening qRT-PCR	541lys	CACMGSNAAYTAYAARACNAA	541–561^*c*^	(9)
	550B	GTRCTCCARTTAGCRCACAT	647–666^*c*^	(9)
	620lyssaC	6-Carboxyfluorescein (FAM)–CAYCAYACHYTVATGACHACHCAYAA–nonfluorescent quencher (QSY)	620–645^*c*^	Modified to include degenerate bases to allow the detection of more diverse lyssaviruses (8)
*Lyssavirus* complete NP PCR	lys001	ACGCTTAACGAMAAA	1–15^*c*^	(9)
	304	TTGACAAAGATCTTGCTCAT	1,514–1,533^*c*^	
*Lyssavirus* complete GP PCR	Lyssa Glyco F	TGGTGYATNAAYATRAAYTC	3,000–3,019^*c*^	(11)
	Lyssa Glyco R	GGRGARTTNARRTTRTARTC	5,520–5,539^*c*^	
*Lyssavirus* NP sequencing primers	lys001	ACGCTTAACGAMAAA	1–15^*c*^	(9)
	550B	GTRCTCCARTTAGCRCACAT	647–666^*c*^	
	304	TTGACAAAGATCTTGCTCAT	1,514–1,533^*c*^	
*Lyssavirus* GP sequencing primers	Lyssa Glyco F	TGGTGYATNAAYATRAAYTC	3,000–3,019^*c*^	(11)
	Sequencing GF1	GAYCCNAGRTAYGARGARTC	3,687–3,706^*c*^	
	Sequencing GF2	ATNCCNGARATGCARTC	4,491–4,507^*c*^	
	Sequencing GF3	CWTCNTGGGARTYNTAYAA	4,849–4,867^*c*^	
	Lyssa Glyco R	GGRGARTTNARRTTRTARTC	5,520–5,539^*c*^	
End verification	Adapt5	ACACTCTTTCCCTACACGACGC	Not applicable	In-house
	nLys5	GGGTCTAGCTTGGCGGC		
	Adapt3	TGACTGGAGTTCAGACGTGTGC		
	nLys3	GCTTGAGTCTGTCCTCCCACTG		

^*a*^ Degenerate bases are indicated using the IUPAC nucleotide code (R = A/G, Y = C/T, S = G/C, W = A/T, K = G/T, M = A/C, B = C/G/T, D = A/G/T, H = A/C/T, V = A/C/G, *N* = A/C/G/T).

^*b*^ Position on human mitochondrial DNA (GenBank accession number: NC012920.1).

^*c*^ Position on Pasteur virus (GenBank accession number: M13215.1).

^*d*^ GP, glycoprotein; NP, nucleoprotein; RT-PCR, reverse-transcriptase PCR; qRT-PCR, quantitative reverse-transcriptase PCR.

**Fig 1:**
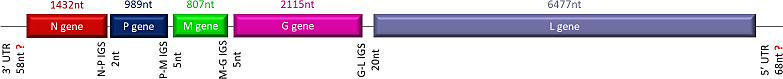
Schematic of PBLV genome organization.

A total of 87.73 million reads with an average read length of 140 bp was obtained. FASTQ files were uploaded to the Galaxy Web platform, and data were analyzed using the server at http://usegalaxy.eu (12). All tools were run for paired-end reads using default parameters unless otherwise noted. FASTQ data sets were quality assessed using FastQC v.0.11.9 (13); reads were quality trimmed (qualified quality Phred score of 20) using fastp v.0.32.2 (14); *de novo* assembly was performed using Megahit v.1.2.9 (15); and contigs were classified using megablast v.2.10.1 (16). A single contig, 12,156 nt in length, was classified as being similar to lyssaviruses and had a nucleotide identity of 73.14% with *Lyssavirus hamburg* [host species: *Eptesicus serotinus* (serotine bat); GenBank accession number: NC009527.1 available at https://www.ncbi.nlm.nih.gov/nuccore/NC_009527] determined using Clustal Omega (17). The 5′ and 3′ ends were verified by amplification of adaptor-ligated DNA fragments using adaptor- and virus-specific primers followed by Sanger sequencing (Table 1), which resolved two misassemblies. Genome annotation was performed using BLASTn and BLASTp (18), and the genome organization was consistent with that of lyssaviruses (Fig. 1). Reads were mapped on the draft genome using Bowtie2 v.2.4.5 (19); duplicate reads were removed and the average sequencing depth was determined, which exceeded 2,600× across the genome, using the SAMtools suite v.1.15.1 (20). The genome was 11,978 nt in length (43.41% GC); however, end verification data suggested that the ends may be longer because, despite repeated attempts, we did not manage to sequence into the adaptors and report the coding-complete genome. At the time of submission, virus isolation attempts had been unsuccessful.

## Data Availability

The phala bat Lyssavirus sequence has been deposited in GenBank under the accession number OQ970171. The version described in this paper is the first version. Raw reads were deposited in the NCBI Sequence Read Archive available at PRJNA971078 under the accession numbers PRJNA971078 (BioProject) and SAMN35019052 (BioSample). The sequence data used for bat identification have been deposited in GenBank under the accession numbers OR096071 (12s rRNA gene) OR091287 (COI gene) and OR105696 (Cytb gene)
